# Improvement of deep learning prediction model in patient‐specific QA for VMAT with MLC leaf position map and patient's dose distribution

**DOI:** 10.1002/acm2.14055

**Published:** 2023-06-01

**Authors:** Ryota Tozuka, Noriyuki Kadoya, Seiji Tomori, Yuto Kimura, Tomohiro Kajikawa, Yuto Sugai, Yushan Xiao, Keiichi Jingu

**Affiliations:** ^1^ Department of Radiation Oncology Tohoku University Graduate School of Medicine Sendai Miyagi Japan; ^2^ Department of Radiology National Hospital Organization Sendai Medical Center Sendai Miyagi Japan; ^3^ Radiation Oncology Center Ofuna Chuo Hospital Kamakura Japan; ^4^ Department of Radiology, Graduate School of Medical Science Kyoto Prefectural University of Medicine Kyoto Japan; ^5^ Department of Radiological Technology Keio University Hospital, Shinjuku Japan

**Keywords:** deep learning, MLC leaf position map, prostate, quality assurance, radiotherapy

## Abstract

**Purpose:**

Deep learning‐based virtual patient‐specific quality assurance (QA) is a novel technique that enables patient QA without measurement. However, this method could be improved by further evaluating the optimal data to be used as input. Therefore, a deep learning‐based model that uses multileaf collimator (MLC) information per control point and dose distribution in patient's CT as inputs was developed.

**Methods:**

Overall, 96 volumetric‐modulated arc therapy plans generated for prostate cancer treatment were used. We developed a model (Model 1) that can predict measurement‐based gamma passing rate (GPR) for a treatment plan using data stored as a map reflecting the MLC leaf position at each control point (MLPM) and data of the dose distribution in patient's CT as inputs. The evaluation of the model was based on the mean absolute error (MAE) and Pearson's correlation coefficient (*r*) between the measured and predicted GPR. For comparison, we also analyzed models trained with the dose distribution in patient's CT alone (Model 2) and with dose distributions recalculated on a virtual phantom CT (Model 3).

**Results:**

At the 2%/2 mm criterion, MAE[%] and *r* for Model 1, Model 2, and Model 3 were 2.32% ± 0.43% and 0.54 ± 0.03, 2.70% ± 0.26%, and 0.32 ± 0.08, and 2.96% ± 0.23% and 0.24 ± 0.22, respectively; at the 3%/3 mm criterion, these values were 1.25% ± 0.05% and 0.36 ± 0.18, 1.57% ± 0.35% and 0.19 ± 0.20, and 1.39% ± 0.32% and 0.17 ± 0.22, respectively. This result showed that Model 1 exhibited the lowest MAE and highest r at both criteria of 2%/2 mm and 3%3 mm.

**Conclusions:**

These findings showed that a model that combines the MLPM and dose distribution in patient's CT exhibited a better GPR prediction performance compared with the other two studied models.

## 1 INTRODUCTION

In recent years, intensity‐modulated radiation therapy (IMRT) and volumetric‐modulated arc therapy (VMAT) have become common treatments at several institutions because of their high precision dose distribution and reduced healthy tissue damage.^1^, ^2^ These methods achieve a highly conformal dose distribution by moving the multileaf collimator (MLC) in complex ways during irradiation. However, because a higher modulation of the MLC introduces uncertainty in dose delivery, performing patient‐specific quality assurance (QA) is recommended to ensure that dose delivery is appropriately administered.^3^, ^4^, ^5^, ^6^, ^7^ Typically, patient‐specific QA is performed by analyzing dose distributions using gamma passing rate (GPR) evaluations based on measurements obtained with film, two‐ and three‐dimensional diode array detectors, or an electronic portal imaging device.^8^, ^9^, ^10^, ^11^ However, these measurement‐based patient‐specific QA methods are problematic because they require extensive labor and time.

Recently, machine learning (ML) and deep learning (DL) have been applied in studies to improve the efficiency of patient‐specific QA by obtaining the GPR prediction without measurements. In ML, Valdes et al. reported virtual IMRT QA; thereafter, several studies have focused on planning complexity for GPR prediction.^12^, ^13^, ^14^, ^15^, ^16^, ^17^ However, although these ML approaches have demonstrated highly accurate predictability, ML‐based prediction requires human intervention for feature extraction, which necessitates knowledge and effort by a well‐trained medical physicist. Furthermore, the features must be re‐extracted when new data is collected, which is time consuming.^18^

To solve this issue, DL, which can make predictions on input data without requiring human intervention, is attracting attention. Interian et al. used fluence maps to train a DL model and predicted GPR without human intervention.^18^ Tomori et al. developed a DL model trained from sagittal dose distributions for GPR prediction.^19^ They further developed a DL model with dummy plans that can be easily retrained at each facility to address differences in treatment planning system modeling and mechanical uncertainty.^20^ Hao et al. showed the possibility of using neural architecture search for optimizing the DL model structure to ensure more efficient and rapid performance without the need for special knowledge.^21^

DL models are evolving to perform faster patient‐specific QA without human intervention, automatically performing everything from feature extraction to model structure. In addition, these models are becoming more robust because they eliminate human bias. However, selecting data to be used as the input remains a manual process; few reports have focused on input data and may not adequately cover the benefits of DL model. In other words, there is room for further development in two major aspects.

First, DL models have the advantage of being able to use various input data because of their high degree of freedom. Nevertheless, only a fluence map and dose distribution are commonly used as input data. This may limit the performance of DL models, thereby requiring further investigation.

Second, several approaches use dose distributions with the plan recalculated on a phantom CT for training. However, this method is time consuming because it requires recalculation on the phantom CT. Furthermore, because the virtual phantoms possess various shapes and characteristics, despite being trained on the same patient data, there may be uncertainty in the DL model depending on the type of phantom. Therefore, using a uniform dose distribution input data easily and quickly would be a better strategy. However, to the best of our knowledge, no report has focused on this aspect in the DL model; further improvement is expected.

To address these issues, we focused on information about MLC leaf position. Previously suggested complexity metrics, such as modulation complexity score (MCS) and MCS for VMAT calculated from aperture area and leaf travel, have been developed and were reported to correlate with GPR.^3^, ^4^, ^5^ These are human‐defined information; however, there may be additional information concealed in the MLC leaf positions during irradiation that could be useful for GPR prediction and cannot be defined by humans. Furthermore, because these are human‐defined one‐dimensional numbers, they do not include the relationship among all MLC leaf positions during irradiation. Therefore, we developed an innovative input data—MLC leaf position map (MLPM)—that includes the relationship between all MLC leaf positions from the beginning to the end of irradiation; we considered extracting useful features for GPR prediction from the MLPM using DL. This facilitates the extraction of human‐definable parameters as well as additional information that cannot be defined by humans from the MLPM, which may contribute to GPR prediction. Furthermore, to facilitate GPR prediction in clinical practice, we developed a model that can directly use the dose distribution calculated on the patient CT for training without requiring the plan to be recalculated on phantom CT. In the present study, we aimed to evaluate the performance of the DL model for predicting GPR from the MLPM and patient dose distribution by comparing our proposed method with previous methods that use phantom dose distribution.

## 2 MATERIALS AND METHODS

### 2.1 Patient datasets

We used 96 single‐arc VMAT plans generated for patients with prostate cancer (target definition, prostate only: 50 pt; prostate with seminal vesicle: 46 pt) undergoing radiotherapy (74 Gy/37 fractions: 48 pt; 70 Gy/28 fractions: 24 pt; 70 Gy/35 fractions: 17 pt; 76 Gy/38 fractions: 5 pt; 65 Gy/26 fractions: 2 pt) in our hospital from July 2018 to May 2021. All plans were created using Eclipse 13.6 treatment planning system (Varian Medical Systems, Palo Alto, USA). The dose calculation algorithm was Acuros XB with a dose grid size of 2 mm. A 10 MV X‐ray beam implemented in TrueBeam STx (Varian Medical Systems) and a high‐definition (2.5 mm) MLC were used. For all GPR measurements, a three‐dimensional diode array detector (ArcCHECK, SunNuclear, Melbourne, USA) was used. Regarding the GPR calculation using SNC Patient (SunNuclear), global GPR for the absolute dose at nine criteria from 1%/1 mm to 3%/3 mm was used (dose threshold: 10%). To ensure the positional accuracy of the setup, all measurements were irradiated within a field of 10 × 10 cm^2^ defined by collimator jaws, which were maintained to have < 1 mm positional error via monthly mechanical QA based on the AAPM Task Group 142 report.^22^ Thereafter, we confirmed that the measured dose profile agreed with the calculated dose profile with a criterion of 0.5 mm. Absolute dose measurements were performed at the isocenter before all measurements to ensure dose accuracy; the discrepancy between calculated and measured dose profiles was confirmed to be within ± 3%.

### 2.2 Creation process of the MLPM

**FIGURE 1:**
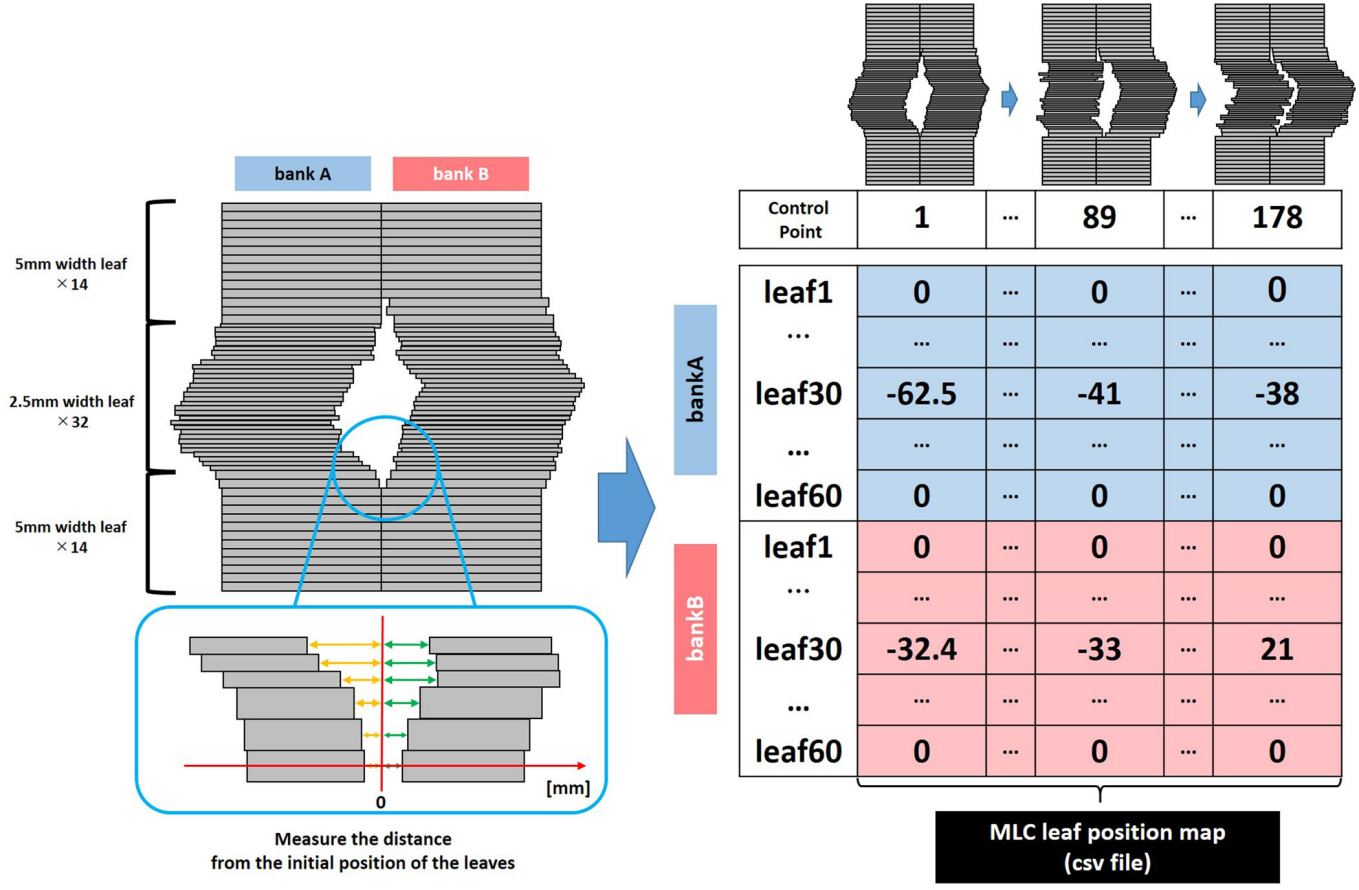
How to make the MLPM from MLC leaves positions per control point. The coordinates when the MLC is closed are 0, and the distance from that point is mapped. In this way, the map can be created reflecting the MLC leaves position for each control point without missing.

To efficiently train the model with information regarding MLC leaf positions during irradiation, the MLPM was generated and added to the input. Figure 1 shows the process of MLPM generation. From the MLC information for each control point, the positions from the first leaf of bank A to the 60^th^ leaf of bank B were extracted and vertically arranged in a row in sequence. By arranging all control points, the MLPM was generated that included all MLC leaf positions per control point during VMAT irradiation. Leaf pairs that were always in closed state in all cases were excluded from the map, assuming that more effective learning could be achieved by removing the parts that did not contain sufficient information.

### 2.3 DL model building and training

**FIGURE 2:**
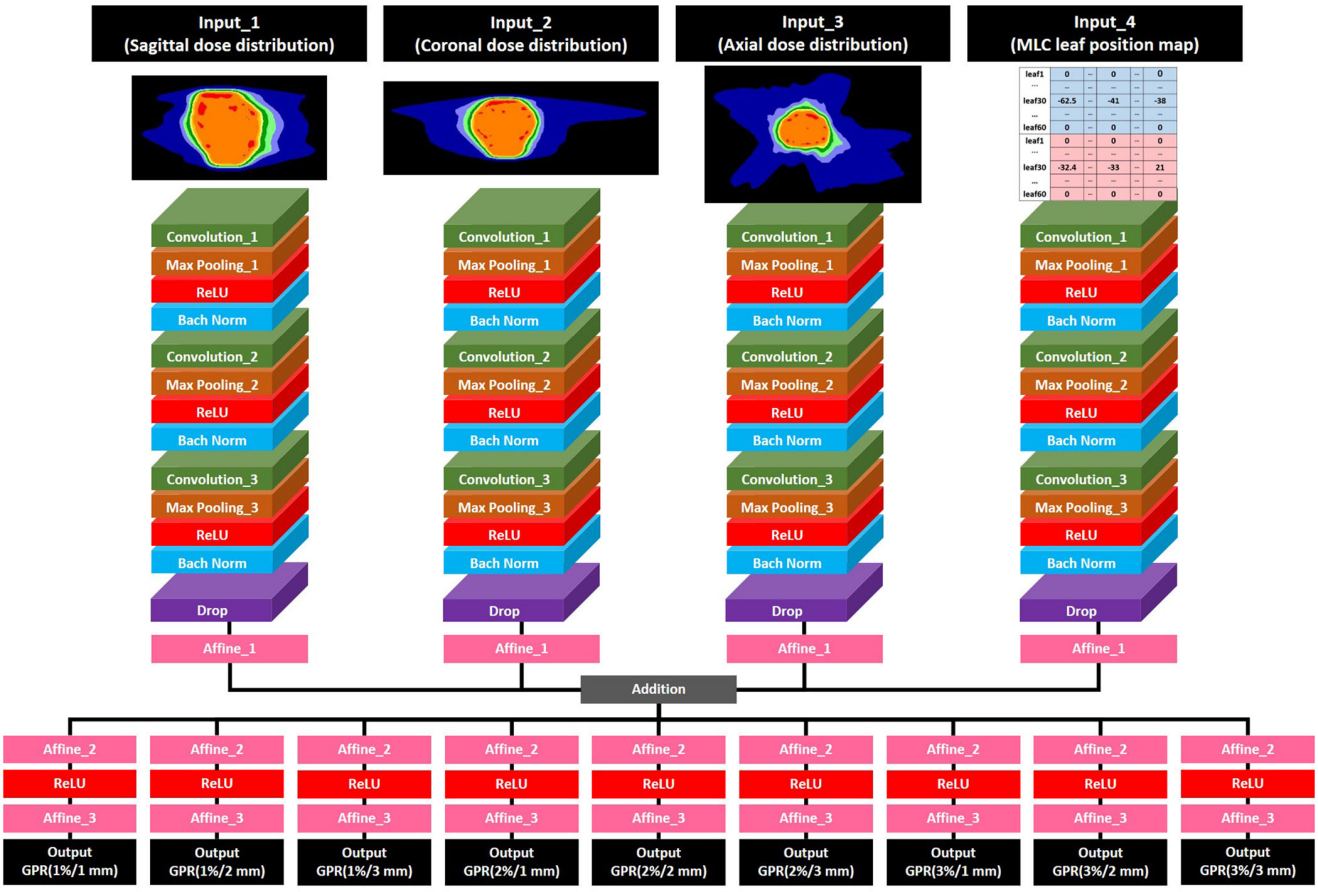
Structure of the original model. Three cross planes extracted from patient's dose distribution and MLPM were used as input, and the output was the GPR at the nine criteria.

Figure 2 shows the model structure; Table 1 presents the detailed hyperparameters at each layer. According to previous research, overfitting occurred when a large number of parameters were trained with a small number of training data; our model had only three convolution layers for each input, thereby making it lighter in weight compared with existing models.^18^ To provide robustness to the GPR criteria, a multioutput model was employed that can simultaneously predict GPRs at nine criteria ranging from 1%/1 mm to 3%/3 mm. In addition, a random drop layer was employed to prevent overfitting; the drop rate was set at 0.3.^23^

**TABLE 1:** Details about hyperparameters at each layer.

Layer	Output	Kernel	Padding	Stride	Number of parameters	Specification
Sagittal dose distribution	68×146×1				0	
Convolution_1	68×146×16	3×3	1×1	1×1	160	
Max Pooling_1	22×48×16	3×3		3×3	0	
Convolution_2	20×46×16	3×3		1×1	2320	
Max Pooling_2	6×15×16	3×3		3×3	0	
Convolution_3	4×13×16	3×3		1×1	2320	
Max Pooling_3	1×4×16	3×3		3×3	0	
Affine_1	20				1300	
Coronal dose distribution	64×200×1	3×3	1×1	1×1	0	
Convolution_1	64×200×16	3×3		3×3	160	
Max Pooling_1	21×66×16	3×3		1×1	0	
Convolution_2	19×64×16	3×3		3×3	2320	
Max Pooling_2	6×21×16	3×3		1×1	0	
Convolution_3	4×19×16	3×3		3×3	2320	
Max Pooling_3	1×6×16				0	
Affine_1	20				1940	
Axial dose distribution	143×242×1				0	
Convolution_1	143×242×16	3×3	1×1	1×1	160	
Max Pooling_1	47×80×16	3×3		3×3	0	
Convolution_2	45×78×16	3×3		1×1	2320	
Max Pooling_2	15×26×16	3×3		3×3	0	
Convolution_3	13×24×16	3×3		1×1	2320	
Max Pooling_3	4×8×16	3×3		3×3	0	
Affine_1	20				10260	
MLC leaves position map	110×178×1				0	
Convolution_1	110×178×16	3×3	1×1	1×1	160	
Max Pooling_1	36×59×16	3×3		3×3	0	
Convolution_2	34×57×16	3×3		1×1	2320	
Max Pooling_2	11×19×16	3×3		3×3	0	
Convolution_3	9×17×16	3×3		1×1	2320	
Max Pooling_3	3×5×16	3×3		3×3	0	
Affine_1	20				4820	
Surface dose distribution	220×680×1				0	
Convolution_1	220×680×16	3×3	1×1	1×1	160	
Max Pooling_1	73×226×16	3×3		3×3	0	
Convolution_2	71×224×16	3×3		1×1	2320	
Max Pooling_2	23×74×16	3×3		3×3	0	
Convolution_3	21×72×16	3×3		1×1	2320	
Max Pooling_3	7×24×16	3×3		3×3	0	
Affine_1	20				53780	
ReLU	20				0	Activation function
Batch Normalization	Same as input				64	
Drop	Same as input				0	Drop rate = 0.3
Affine_2	20				420	
Affine_3	1				21	
Addition	20				0	

An open platform (Neural Network Console, Sony, Japan) was used to build and train the model, with the number of epochs set to 200, batch size to 4, optimization algorithm to Adam, and learning rate to 0.001. The values of the weights and biases were adopted at the epochs with the smallest errors in the validation data.

All data were divided into training and test data at a ratio of 3:1, with 20% of the training data used as validation data. To improve generalization performance, a four‐fold cross‐validation was conducted. In each partition, the mean absolute error (MAE) and Pearson's correlation coefficient (*r*) of the measured GPR and predicted GPR of the test data were calculated and evaluated based on the average of the four times.

### 2.4 Verification of input data accuracy by model patterns

**FIGURE 3:**
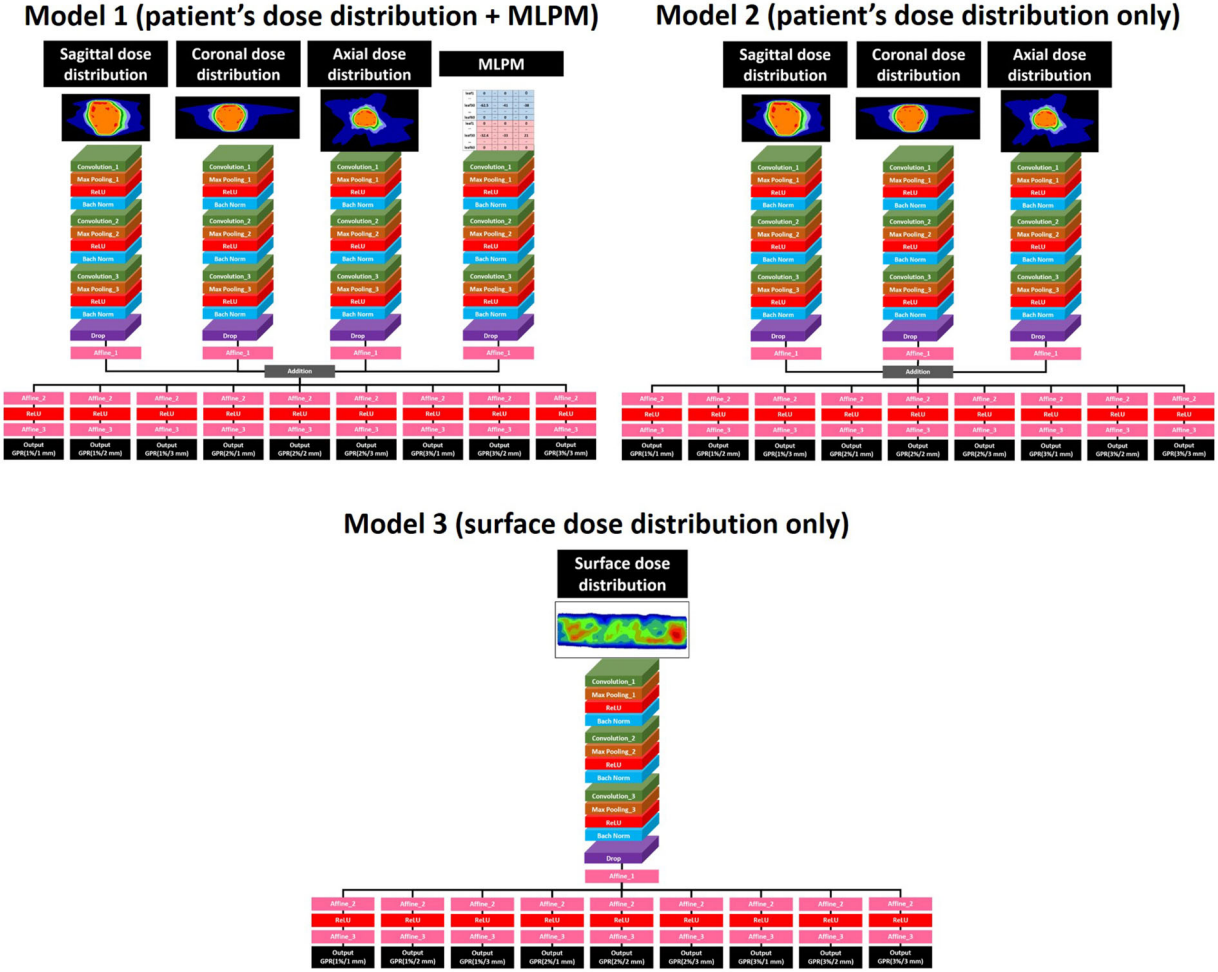
Outline of the three models. Three models with different input data were tested to confirm the dependence of prediction accuracy on input data.

To compare the usefulness of our model (Model 1: three planes of patient dose distribution through the isocenter + the MLPM as inputs), two additional models were built: Model 2 (only three planes of patient dose distribution through the isocenter as input) and Model 3 (surface dose distribution as input) (Figure 3). Using these comparisons, we believed that it would be possible to determine the input data that contributed the most to prediction accuracy. To ensure a fair comparison, the same hyperparameters and datasets were used for all models.

## 3 RESULTS

**FIGURE 4:**
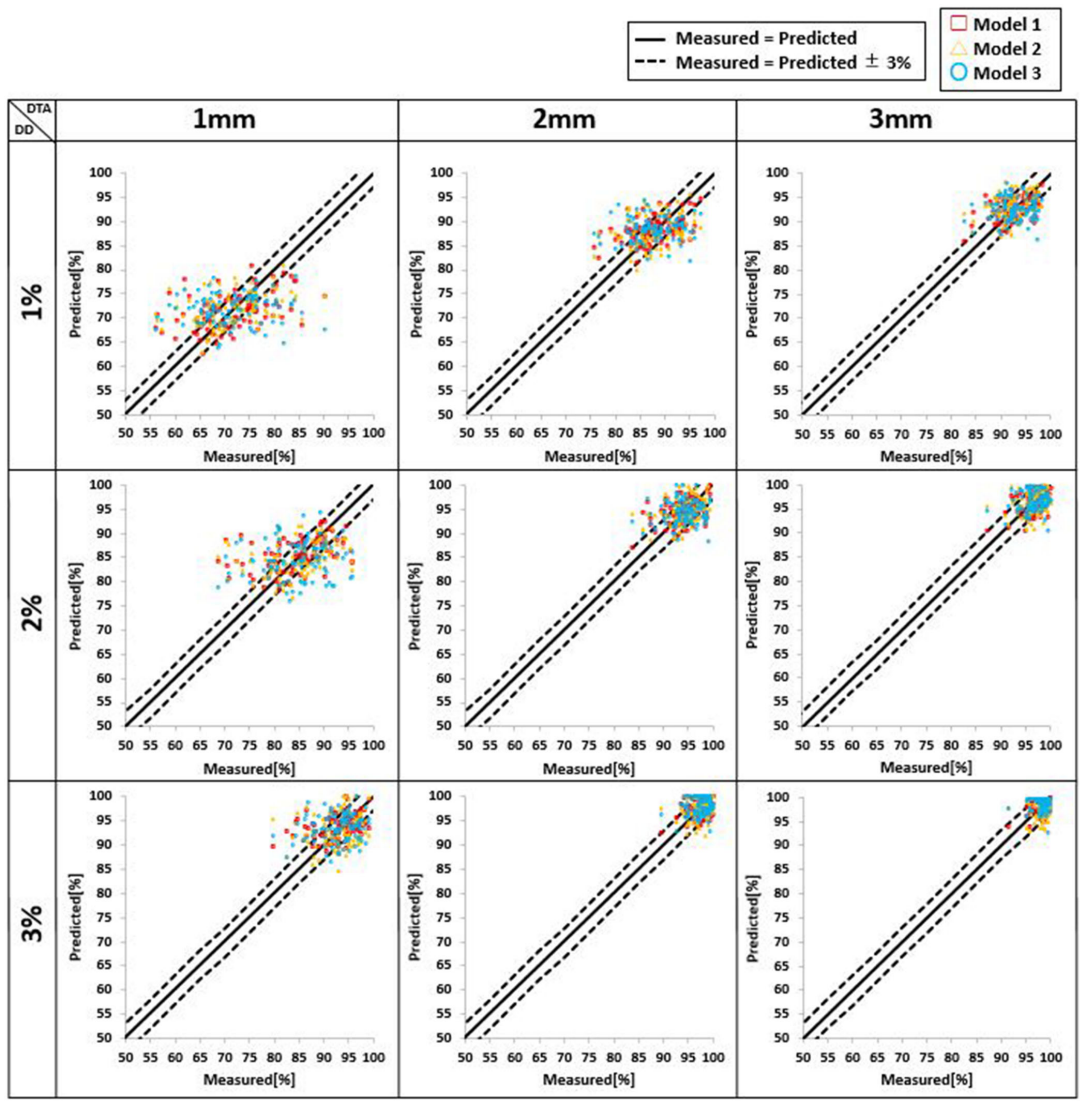
Scatter plot comparing measured vs predicted GPR (%) by dose distribution (DD) and distance to agreement (DTA). The solid line is the line where the measured and predicted GPRs are of the same value, and the dotted line is the line where the error between the measured and predicted GPRs is at ± 3%. The results for Model 1, Model 2, and Model 3 are plotted by red square, yellow triangle, and blue circle, respectively.

Scatter plots of measured and predicted GPRs for the three models are shown in Figure 4; MAE and *r* results are presented in Table 2. Regarding the MAE, the mean value at all criteria was 2.72% ± 1.14%, 3.08% ± 1.16%, and 3.36% ± 1.48% for Model 1, Model 2, and Model 3, respectively, showing that Model 1 had the lowest mean value among all three models. Furthermore, Model 1 exhibited the lowest MAE at all criteria (e.g., at the 3%/2 mm criterion: Model 1, 1.57%; Model 2, 1.84%; and Model 3, 1.85%). Regarding the *r*, the mean value at all criteria was 0.45 ± 0.13, 0.26 ± 0.14, and 0.22 ± 0.19 for Model 1, Model 2, and Model 3, respectively, showing that Model 1 exhibited the highest mean value among all three models. Furthermore, Model 1 had the highest *r* at all criteria (e.g., at the 3%/2 mm criterion: Model 1, 0.44; Model 2, 0.19; and Model 3, 0.20).

**TABLE 2:** MAE and *r* results at each criterion for the three models.

Model	γ criteria	MAE ± SD	*r* ± SD
Model 1	1%/1 mm	4.88 ± 0.31	0.43 ± 0.14
	1%/2 mm	3.15 ± 0.31	0.51 ± 0.06
	1%/3 mm	2.36 ± 0.17	0.50 ± 0.08
	2%/1 mm	4.00 ± 0.21	0.42 ± 0.12
	2%/2 mm	2.32 ± 0.43	0.54 ± 0.03
	2%/3 mm	1.86 ± 0.34	0.51 ± 0.14
	3%/1 mm	3.06 ± 0.17	0.36 ± 0.12
	3%/2 mm	1.57 ± 0.12	0.44 ± 0.11
	3%/3 mm	1.25 ± 0.05	0.36 ± 0.18
Model 2	1%/1 mm	4.93 ± 0.42	0.34 ± 0.13
	1%/2 mm	3.72 ± 0.51	0.28 ± 0.11
	1%/3 mm	2.68 ± 0.17	0.32 ± 0.06
	2%/1 mm	4.46 ± 0.27	0.26 ± 0.12
	2%/2 mm	2.70 ± 0.26	0.32 ± 0.08
	2%/3 mm	2.10 ± 0.27	0.27 ± 0.12
	3%/1 mm	3.68 ± 0.22	0.15 ± 0.17
	3%/2 mm	1.84 ± 0.28	0.19 ± 0.11
	3%/3 mm	1.57 ± 0.35	0.19 ± 0.20
Model 3	1%/1 mm	5.67 ± 0.63	0.19 ± 0.12
	1%/2 mm	3.95 ± 0.78	0.25 ± 0.23
	1%/3 mm	3.09 ± 0.33	0.21 ± 0.21
	2%/1 mm	5.49 ± 0.62	0.24 ± 0.10
	2%/2 mm	2.96 ± 0.23	0.24 ± 0.22
	2%/3 mm	2.19 ± 0.14	0.21 ± 0.25
	3%/1 mm	3.62 ± 0.11	0.27 ± 0.09
	3%/2 mm	1.85 ± 0.11	0.20 ± 0.21
	3%/3 mm	1.39 ± 0.32	0.17 ± 0.22

At the 3%/2 mm criterion, 84.4%, 82.3%, and 81.3% of all predicted GPRs for Model 1, Model 2, and Model 3, respectively, were within ± 3% of measured GPRs. Furthermore, at the 3%/3 mm criterion, 93.8%, 84.4%, and 90.6% of all predicted values for Model 1, Model 2, and Model 3, respectively, were within ± 3% of the measured GPRs. These results showed that compared with Model 2 and Model 3, a higher percentage of predicted values within ± 3% at both criteria was observed for Model 1.

## 4 DISCUSSION

We hypothesized that MLC leaf position at each control point would provide useful information for GPR prediction by the DL model. The MLPM was developed by mapping MLC leaf positions. For more efficient and robust prediction, a model that can predict GPR from the dose distribution in patient's CT without recalculation on phantom CT was developed. The model that combined data of the MLPM and the dose distribution in patient's CT (Model 1) was more accurate compared with the conventional model for GPR prediction that uses dose distribution recalculated on phantom CT.

Among the three models, Model 1 exhibited the lowest mean MAE and highest mean *r*. Particularly, the prediction accuracy was improved for cases that showed large prediction errors with the conventional model. The model trained using only the dose distribution in patient's CT without the MLPM (Model 2) unexpectedly achieved only the same level of accuracy as the conventional model. The difference in accuracy observed between Model 1 and Model 2 may be attributed to the combination of the dose distribution in patient's CT and MLPM, which provided additional information, used in Model 1 compared with dose distribution data alone in Model 2. Hirashima et al. showed that an ML model combining dosiomics and plan complexity metrics improved the prediction accuracy.^17^ Tomori et al. demonstrated that providing the model with information regarding MU values, planning target volume, and organ‐at‐risk volume in addition to the dose distribution improved the accuracy compared with the model using only the dose distribution.^19^ Their result is consistent with our findings. Therefore, combining multiple input data may be important because additional information besides the dose distribution in patient's CT can be provided to the model to supplement information that cannot be obtained from the dose distribution alone.

Several studies have stated that criteria such as those used in clinical practice (e.g., 3%/2 mm or 3%/3 mm) do not sufficiently distribute the GPR and should thus be compared with GPRs predicted at stringent criteria.^19^, ^20^, ^24^ Therefore, at loose criteria such as 3%/2 mm or 3%/3 mm, most measured GPRs were close to 100%; thus, although the model does not learn the feature, it will only produce an unnecessarily high value, thereby resulting in an apparently good MAE. To solve this issue, obtaining GPR prediction at multiple criteria and evaluating them using the correlation coefficient are essential. The correlation coefficient cannot be high simply owing to a high GPR prediction; it must appropriately capture the large and small relationship of the GPRs for each case, and the characteristics of each case must be accurately learned. Compared with the results obtained using Model 2 and Model 3 at the 1%/1 mm criterion, the ones obtained using Model 1 at the 3%/3 mm criterion exhibited the lowest MAE and highest *r*. This indicates that the MLPM enabled the model to learn sufficient features in an efficient manner despite the 3%/3 mm criterion.

Clinically relevant criteria for conventional fractionation are between 2−3% and 2−3 mm. Our model exhibited good prediction accuracy in this range (e.g., 3%/2 mm proposed in AAPM Task Group 218 report).^4^ Meanwhile, the 1 mm region across all dose difference criteria showed a slightly lower MAE prediction accuracy. Several studies have reported that more stringent criteria (e.g., 2%/1 mm) may be useful for stereotactic radiotherapy owing to a higher sensitivity in the detection of delivery errors. Therefore, our model requires improvement to increase the MAE prediction accuracy in this range.^25^, ^26^

The present study has several limitations. First, the number of data used was small, and the predictions may be unstable. Therefore, further validation is necessary to increase the number of cases. Second, the data used in this study was based on a single institution, device, and treatment site. To increase the robustness of the model, data collected from other institutions, devices, and various treatment sites should be used as input to validate the generalization performance. To utilize the DL model in clinical practice, obtaining predictions from onsite and online information is important to perform virtual QA more efficiently and within less duration. Third, we used single‐arc plans in this study. Meanwhile, complex plans with two or more arcs are also used in clinical practice. Shen et al. showed that two‐arc plans were more complex than single‐arc plans because plans created with two arcs facilitate the use of smaller and/or more irregular MLC apertures compared with a plan created with only a single arc.^27^ For example, to predict GPR with two‐arc plans, it is necessary to train a model with two‐arc plans. We plan to examine whether our method can be extended in this regard in the future. Fourth, different planning optimization strategies (e.g., use of an avoidance sector and avoidance structure function) may result in different types of plan complexity.^28^ In such cases, our model may have a larger prediction error. Fifth, our model predicts only the GPR and not the measured dose distribution. To improve the accuracy further, there may be a need to predict the spatial dose distribution. Sixth, we only employed gamma evaluation for prediction. This method is often used in previous reports and recommended in AAPM Task Group reports 119 and 218. Although it has been stated that GPR provides insight into the overall system errors, it does not correlate with dose difference or dose volume histogram.^6^, ^7^, ^29^, ^30^ A deeper consideration of the reliability of virtual QA results requires cross‐validation, for instance, by combining absolute point dose prediction, which would be a topic for future research.

The MLPM can be easily retrieved from the treatment plan. Furthermore, even faster prediction can be achieved by employing the dose distribution in patient's CT for training, thereby eliminating the need for the recalculation of dose distribution in the phantom CT. As the model developed in this study combines the abovementioned factors, it may efficiently predict GPR and help reduce the measurement burden in patient‐specific QA.

## 5 CONCLUSION

We developed a DL model that predicts GPR based on a combination of a map reflecting MLC leaf positions and the dose distribution in patient's CT as inputs. Our result showed that a model that combines the MLPM and dose distribution in patient's CT exhibited better GPR prediction performance compared with the other two studied models.

## AUTHOR CONTRIBUTIONS

Ryota Tozuka, Noriyuki Kadoya, Seiji Tomori, Yuto Kimura, Tomohiro Kajikawa, and Yuto Sugai contributed to the conception and design of the study. Ryota Tozuka, Noriyuki Kadoya, and Yushan Xiao performed the analysis. Ryota Tozuka mainly drafted the manuscript. Noriyuki Kadoya and Keiichi Jingu reviewed the manuscript. All authors read and approved the final manuscript.

## CONFLICT OF INTEREST STATEMENT

Dr. Kadoya had the stock of AiRato.Inc.
